# Associations of *CFH* Polymorphisms and *CFHR1-CFHR3* Deletion with Blood Pressure and Hypertension in Chinese Population

**DOI:** 10.1371/journal.pone.0042010

**Published:** 2012-07-25

**Authors:** Wei Gan, Johnna Wu, Ling Lu, Xu Xiao, Heng Huang, Fulong Wang, Jingwen Zhu, Liang Sun, Gang Liu, Yi Pan, Huaixing Li, Xu Lin, Yan Chen

**Affiliations:** 1 Key Laboratory of Nutrition and Metabolism, Institute for Nutritional Sciences, Shanghai Institutes for Biological Sciences, Chinese Academy of Sciences, Graduate School of the Chinese Academy of Sciences, Shanghai, China; 2 Department of Emergency Medicine, The Sichuan Provincial People’s Hospital, Chengdu, China; Kunming Institute of Zoology, Chinese Academy of Sciences, China

## Abstract

Dysregulation of the complement system has been linked to pathogenesis of hypertension. However, whether genetic changes of complement factor H (CFH) and its related genes are associated with hypertension is unknown. We genotyped three SNPs in the *CFH* gene cluster that are closely linked to age-related macular degeneration, namely rs1061170 (Y402H), rs2274700 (A473A) and rs7542235 (*CFHR1–3*Δ), and tested for their associations with blood pressure and hypertension risk in a population-based cohort including 3,210 unrelated Chinese Hans (50–70 years of age) from Beijing and Shanghai. We found that rs2274700 (A473A) and rs7542235 (*CFHR1–3*Δ) were both significantly associated with diastolic blood pressure (DBP) (β = 0.632–1.431, *P*≤0.038) and systolic blood pressure (SBP) (β = 1.567–4.445, *P*≤0.008), and rs2274700 (A473A) was associated with hypertension risk (OR [95%CI]: 1.175 [1.005–1.373], *P* = 0.048). Notably, the associations of rs2274700 (A473A) with DBP (*P* = 2.1×10^−3^), SBP (*P* = 8×10^−5^) and hypertension risk (*P* = 7.9×10^−3^) were significant only in the individuals with low CRP levels (<2.0 mg/l), but not in those with CRP levels ≥2.0 mg/l (*P*≥0.0807) (*P* for interaction ≤0.0467). However, no significant association between rs1061170 (Y402H) and blood pressure or hypertension risk was observed (*P*≥0.259). In conclusion, our results suggest that genetic variations in *CFH* and its related genes may contribute to hypertension risk in Chinese Hans.

## Introduction

Hypertension has been widely considered to be a multifactorial inflammatory disease. Growing evidence suggests that chronic low-grade activation of inflammation and immune system plays active roles in the pathogenesis of hypertension [1]. Elevated levels of circulating complement component 3 (C3), which plays a central role in the innate immune system, has been associated with increased risk of hypertension, metabolic syndrome, type 2 diabetes, and cardiovascular diseases [2]–[6]. C3 can be activated by all three complement pathways. Each complement cascade results in cleavage of C3 and generation of its functional peptide, which further initiates a massive amplification of further cleavages of other complement components. Such cleavage and amplification is controlled by complement factor H (CFH) and its related proteins [7]. However, whether dysfunction or mutation of CFH and its related proteins is associated with hypertension is currently unknown.

**Table 1:** Characteristics of the study samples.

Characteristics	Beijing	Shanghai	All
N^a^ (% men)	1574(45.2)	1636(43.5)	3210(44.3)
Age^b^ (years)	58.3±5.9	58.9±6.0	58.6±6.0
BMI^b^ (kg/m^2^)	25.2±3.7	23.6±3.3	24.4±3.6
SBP^b^ (mmHg)	147.5±24.4	138.6±23.8	143.0±24.5
DBP^b^ (mmHg)	82.8±11.5	80.5±11.6	81.6±11.6
hs-CRP^c^ (mg/l)	0.84(0.79, 0.89)	0.60(0.56, 0.63)	0.71(0.68, 0.73)
Normotension^a^ (%)	182 (11.5)	368 (22.5)	550 (17.1)
Hypertension^a^ (%)	977 (62.1)	804 (49.1)	1781 (55.5)
Taking antihypertensive^a^ (%)	436 (27.7)	461 (28.2)	897 (27.9)

Values are expressed as ^a^number (percentage), ^b^means ± SD or ^c^geometric mean (95%CI). BMI indicates body mass index, SBP indicates systolic blood pressure DBP indicates diastolic blood pressure, hs-CRP indicates high-sensitivity C-reactive protein.

The human CFH and five CFH-related proteins (CFHR1–5) are all secreted plasma glycoproteins, which are primarily synthesized in the liver and play similar role in complement control. They all contain multiple short consensus repeat (SCR) domains that can protect host cells against complement damage, some of which serve as functional C-reactive protein (CRP)-binding sites in acute phase concentrations [8]. The genes encoding CFH and its five related proteins (CFHR1–5) all lie in tandem within a 355 kb genomic region (1q32). The genome-wide association studies showed that the common variants in *CFH* and *CFH*-related genes (*CFHR1–5*), and the haplotypes formed by these risk variants are significantly associated with the risk of age-related macular degeneration (AMD) [9]–[13]. The major risk variants with plausibly functional relevance include a nonsynonymous coding variant rs1061170 (Y402H), a synonymous coding variant rs2274700 (A473A), and a common deletion variant spanning *CFHR1* and *CFHR3* genes (*CFHR1–3Δ*), which is tagged by the G-allele of SNP rs7542235. It has been proposed that the rs1061170 (Y402H) polymorphism affects the binding affinity to C-reactive protein or heparin, and hinders the function of CFH in regulating the complement pathway [14]. Consistent with such an observation, available evidence suggests that the serum CRP levels and the rs1061170 (Y402H) variant of the *CFH* gene have synergistic effects on the risk of AMD [15]. On the other hand, the synonymous coding variant rs2274700 (A473A) is located at CFH-CRP binding site SCR-6/8 and may be in linkage disequilibrium (LD) with a genetic variant that affect expression or function of CFH or binding of the CRP to CFH.

In this population-based cohort study of Chinese Hans, we examined whether the previously reported AMD-associated SNPs in the region of *CFH* gene cluster are associated with blood pressure or hypertension, and whether there is an interaction effect between plasma CRP levels and genotypes of these AMD-associated SNPs on blood pressure and hypertension risk.

**Table 2:** Associations of *CFH* genetic variants with blood pressure and hypertension in Chinese Hans.

SNP	Risk allele/Alternative	RAF	DBP(mmHg)		SBP(mmHg)		HTN			
							RAF		OR (95%CI)	*P*
			β(SE)	*P*	β(SE)	*P*	Case = 1781	Control = 550		
rs1061170 (Y402H)^a^	C/T									
Beijing (n = 1558)		0.071	1.334(0.880)	0.130	2.283(1.740)	0.19	0.073	0.068	1.004(0.602–1.673)	0.989
Shanghai (n = 1612)		0.047	−0.046(1.034)	0.964	0.581(1.978)	0.769	0.047	0.050	1.045(0.655–1.669)	0.853
All^b^ (n = 3170)		0.059	0.754(0.670)	0.303	1.541(1.306)	0.259			1.026(0.727–1.449)	0.887
*P*_(heter)_				0.309		0.518				0.908
**rs2274700 (A473A)**	T/C									
Beijing (n = 1544)		0.442	0.214(0.433)	0.621	1.427(0.855)	0.095	0.449	0.427	1.141(0.893–1.460)	0.292
Shanghai (n = 1584)		0.420	1.044(0.430)	0.015	1.698(0.825)	0.040	0.432	0.400	1.197(0.978–1.466)	0.081
All^b^ (n = 3128)		0.431	0.632(0.305)	0.038	1.567(0.594)	0.008^c^			1.175(1.005–1.373)	0.048
*P*_(heter)_				0.174		0.820				0.770
**rs7542235 (** ***CFHR1–3*** **Δ)** a	G/A									
Beijing (n = 1544)		0.076	0.681(0.858)	0.428	4.211(1.696)	0.013	0.079	0.070	1.284(0.774–2.131)	0.334
Shanghai (n = 1584)		0.067	2.239(0.891)	0.012	4.684(1.712)	0.006^c^	0.072	0.060	1.180(0.776–1.795)	0.439
All^b^ (n = 3128)		0.072	1.431(0.618)	0.019	4.445(1.205)	0.0002^c^			1.221(0.884–1.687)	0.219
*P*_(heter)_				0.208		0.844				0.801

β (SE) and OR (95%CI) were expressed as changes in blood pressure and odds of hypertension for increasing copy of risk alleles in Beijing, Shanghai and total population (All). *P* values were adjusted for age, sex and BMI.

a Dominant model was applied, otherwise additive model was used.

b β (SE) and OR (95%CI) were calculated using an inverse-variance weighted (fixed effect) meta-analysis and *P* values were calculated using a z-score weighted approach.

c The associations remained significant after Bonferroni correction for multiple tests (the Bonferroni corrected cutoff *P* value was 0.05/6 = 0.0083). RAF: Risk Allele Frequency, SBP: Systolic Blood Pressure, DBP: Diastolic Blood Pressure, HTN: Hypertension, *P*_(heter)_ : *P* for heterogeneity.

## Materials and Methods

### Study Population

The study sample consisted of 3,210 unrelated Han Chinese enrolled in a population-based cohort originally designed to study the nutrition and health of the aging population (50–70 years). The study design and data collection has previously been described in detail [16]. Briefly, all participants were unrelated and with at least 20 years’ residence in Beijing or Shanghai. A multistage sampling method was used to recruit the study participants. Two urban districts and one rural district were chosen to represent a range of socioeconomic groups. In the sampling process, 400 participants from each urban district and 800 persons for each rural district were randomly selected from eligible candidates in the residential registration record. One person from each household was included in the study. The field work was conducted simultaneously in both Beijing and Shanghai from March to June 2005. All participants were asked to fast overnight before attending a physical examination, which included standard anthropometric measurements. A blood sample was collected and questionnaires on medical history, nutrition, and physical activity were completed. The sitting blood pressure of the subjects was measured by an Omron HEM-705CP Blood Pressure Monitor (OMRON Healthcare Inc., Vernon Hills, Illinois) three times after the subject being seated for 5 min and the average of the last two measurements was used for the analyses. For individuals taking anti-hypertensive medication, 10 mmHg and 5 mmHg were added to the observed values of systolic blood pressure (SBP) and diastolic blood pressure (DBP) respectively to account for the treatment effects [17]. BMI was calculated as weight (kg)/height (m)^2^. Plasma high-sensitivity C-reactive protein (hs-CRP) was measured by a particle-enhanced immunoturbidimetric assay (Ultrasensitive CRP kit, Orion Diagnostica, Espoo, Finland) using micro particles coated with anti-human CRP antibodies. CRP levels were classified as low or high according to the cutoff of 2.0 mg/l which was suggested to be an effective predictor for CVD in Chinese older population [18]. Hypertension (HTN) was defined by whether the subjects had SBP ≥140 mmHg and/or DBP ≥90 mmHg, or had previously been diagnosed with hypertension and/or was taking anti-hypertensive medication. The study protocol was approved by the Institutional Review Board of the Institute for Nutritional Sciences, Shanghai Institutes for Biological Sciences, Chinese Academy of Sciences and all participants provided their written consents. Phenotypic characteristics of the population are shown in Table 1.

**Table 3:** Associations of *CFH* genetic variants genotypes with blood pressure and hypertension stratified by C-reactive protein levels.

SNP genotype	DBP (mmHg)		SBP (mmHg)		Hypertension	
	Mean ± SE	*P*	Mean ± SE	*P*	OR(95%CI)	*P*
**rs1061170 (Y402H)**						
Low CRP levels (<2.0 mg/l, n = 2607)						
TT	82.43±0.25		142.89±0.49		1.00 (reference)	
CT + CC	82.99±0.71		144.11±1.38		1.00 (0.69–1.45)	
Dominant	0.59 (0.75)	0.4304	1.13(1.46)	0.4383	1.00 (0.69–1.45)	0.9930
Elevated CRP levels (≥2.0 mg/l, n = 603)						
TT	86.59±0.55		150.89±1.09		1.00 (reference)	
CT + CC	87.81±1.39		153.30±2.75		1.01 (0.37–2.74)	
Dominant	1.22 (1.46)	0.4049	2.16(2.85)	0.4487	1.01 (0.37–2.74)	0.9891
*P* for interaction		0.8126		0.7867		0.7579
**rs2274700 (A473A)**						
Low CRP levels (<2.0 mg/l, n = 2607)						
CC	81.28±0.42		140.21±0.81		1.00 (reference)	
CT	82.94±0.33		144.04±0.64		1.42 (1.09–1.84)	
TT	83.15±0.55		145.00±1.07		1.22 (1.03–1.45)	
Additive	1.04 (0.34)	0.0021	2.58 (0.66)	0.00008	1.26 (1.06–1.49)	0.0079
Elevated CRP levels (≥2.0 mg/l, n = 603)						
CC	86.69±0.86		151.78±1.69		1.00 (reference)	
CT	87.67±0.74		152.52±1.45		1.44 (0.69–3.02)	
TT	84.12±1.16		145.39±2.27		0.74 (0.49–1.11)	
Additive	−0.95 (0.70)	0.1903	−2.46 (1.36)	0.0807	0.82 (0.53–1.25)	0.3352
*P* for interaction		0.0110		0.0010		0.0467

Data are means ± SE, OR (95%CI) or β (SE). β (SE) and OR (95%CI) were calculated using an inverse-variance weighted (fixed effect) meta-analysis and *P* values were calculated using a z-score weighted approach. *P* for interaction was calculated using general linear regression after adjusting for age, sex and BMI.

### Genotyping

Genomic DNA was extracted from peripheral blood leukocytes by a salting-out procedure (http://protocol-online.org/prot/Detailed/3171.html). Three AMD-associated SNPs [9], [19]–[22], namely rs1061170 (Y402H), rs2274700 (A473A) and rs7542235 (*CFHR1–3Δ*), were genotyped with TaqMan SNP allelic discrimination by the ABI PRISM 7900HT sequence detection system (Applied Biosystems) according to manufacturer’s protocol. In brief, polymerase chain reaction was conducted by using a TaqMan SNP genotyping master mix and TaqMan SNP genotyping assay (Applied Biosystems). The allelic-specific fluorescence was detected by the ABI PRISM 7900HT sequence detection system after amplification. The allelic discrimination was determined by using the software SDS2.3 (Applied Biosystems). The genotyping success rate was ≥97.4% and the concordance rate was ≥99% based on 12% duplicate samples (n = 384) for each SNPs. The genotype distributions of all the three SNPs were in Hardy-Weinberg equilibrium (*P*≥0.32), and those for rs2274700 (A473A) and rs7542235 (*CFHR1–3Δ*) were found to be similar between Beijing and Shanghai participants (*P*≥0.1909), but significantly different for rs1061170 (Y402H) (*P*<0.0001).

### Statistical Analyses

The Hardy-Weinberg equilibrium and genotype distributions between Beijing and Shanghai participants were tested using likelihood ratio test. The phenotype differences between the participants from Beijing and Shanghai were analyzed by the Student’s t-test or χ^2^ test where appropriate. Association analyses for all SNPs were performed in the Beijing and Shanghai subpopulations separately. Subsequently, summary statistics (beta, se, OR and 95%CI) were calculated using an inverse-variance weighted (fixed effect) meta-analysis and *P* values were calculated using a z-score weighted approach. The association analysis assumed an additive genetic model for SNP rs2274700 (A473A), and a dominant genetic model for SNP rs1061170 (Y402H) and rs7542235 (*CFHR1–3Δ*) with low minor allele frequencies (counts of minor allele homozygote <20). A generalized linear regression model was used to test associations of each SNP with blood pressure. Logistic regression was used to examine association between each SNPs and risk of hypertension. Cochran’s Q test was applied to assess heterogeneity among different groups. All association analyses were adjusted for age, sex, and BMI. The potential modifying effects of CRP levels on genetic associations with blood pressure or risk of hypertension were evaluated by introducing a CRP categories × gene term into the linear or logistic regression models with adjustment for age, sex and BMI. Linkage disequilibrium was estimated using Haploview V3.2 (http://www.broad.mit.edu/mpg/haploview). All *P*-values were nominal and two-sided. Association analyses were performed with SAS version 9.2 (SAS Institute, Cary, NC, USA).

## Results

The three AMD-associated SNPs genotyped in this study, namely the rs1061170 (Y402H), rs2274700 (A473A) and rs7542235 (*CFHR1–3Δ*) are all in low linkage disequilibrium with each other (r^2^≤0.09). The observed minor allele frequencies of rs1061170 (Y402H) and rs7542235 (*CFHR1–3Δ*) (0.059 and 0.072 respectively) in our population were comparable to those in HapMap-CHB samples (0.067 and 0.089 respectively), but significantly lower than HapMap-CEU samples (0.282 and 0.230 respectively).

We first examined the association of each SNP with blood pressure and hypertension. As shown in Table 2, blood-pressure-increasing alleles of rs2274700 (A473A) and rs7542235 (*CFHR1–3Δ*) were both significantly associated with higher DBP (*P*≤0.015) and SBP (*P*≤0.04) in the Shanghai subpopulation, and that of rs7542235 (*CFHR1–3Δ*) was also associated with higher SBP in the Beijing subpopulation (*P* = 0.013). However, we found no evidence for association of rs1061170 (Y402H) with DBP or SBP (*P*≥0.130). When we combined data from the two sub-populations, the blood-pressure-increasing alleles of rs2274700 (A473A) and rs7542235 (*CFHR1–3Δ*) were both significantly associated with higher DBP (rs2274700: β = 0.632, *P* = 0.038; rs7542235: β = 1.431, *P* = 0.019) and SBP (rs2274700: β = 1.567, *P* = 8×10^−3^; rs7542235: β = 4.445, *P* = 2×10^−4^). The rs2274700 (A473A) blood-pressure-increasing allele also showed an association with increased risk of hypertension (OR: 1.175 [1.005–1.373], *P* = 0.048). There was no significant heterogeneity of associations for those SNPs between the Beijing and Shanghai populations (*P*≥0.174). However, only the associations with SBP remained significant after Bonferroni correction for the multiple tests (the *P* value cutoff for Bonferroni correction was 0.05/6 = 0.0083). The relative contributions of SNPs rs2274700 (A473A) and rs7542235 (*CFHR1–3Δ*) to the variation in SBP were 0.17% and 0.35%, respectively.

Since rs1061170 (Y402H) and rs2274700 (A473A) are both located at the CRP-binding site in CFH, we therefore tested whether there was an interaction between plasma CRP levels and genotypes of these two SNPs for association with blood pressure or hypertension risk. As shown in Table 3, the associations of rs2274700 (A473A) with DBP (*P* = 2.1×10^−3^) and SBP (8×10^−5^), and hypertension risk (7.9×10^−3^)were significant only in individuals with low CRP levels (<2.0 mg/l), but not in those with elevated CRP levels (≥2.0 mg/l) (*P*≥0.0807, *P* for interaction: 0.001–0.047). No significant interaction was observed between rs1061170 (Y402H) genotype and plasma CRP level for association with blood pressure or hypertension (*P*≥0.7579) (Table 3).

## Discussion

In this population-based cohort study of Chinese Hans, we demonstrated that rs2274700 (A473A) and rs7542235 (*CFHR1–3Δ*) were both significantly associated with blood pressure. Notably, the associations of rs2274700 (A473A) with blood pressure was significant only in the individuals with low CRP levels (<2.0 mg/l), but not in those with elevated CRP levels (≥2.0 mg/l). However, no significant association between rs1061170 (Y402H) and blood pressure and hypertension risk was observed.

To our knowledge, this is the first study to provide evidence that genetic variants in *CFH* and *CFHR1/R3* are associated with blood pressure, in accordance with previous observations that increased plasma C3 levels may contribute to hypertension risk [3], [19], [23]–[26]. Although the molecular mechanism by which the rs2274700 (A473A) and rs7542235 (*CFHR1–3Δ*) variants contribute to hypertension risk remains unknown, a possible explanation is that the mutations caused by A473A and CFHR1–3Δ may lead to changes in expression or function of CFH and CFHR1/R3, and consequently result in dysregulation of complement system and inflammation. Consistent with this notion, CFHR1 and CFHR3 deficiency resulting from the CFHR1–3Δ has been associated with complement over-activation, increased inflammatory response, and increased risks of inflammation-related diseases, such as atypical hemolytic uremic syndrome (aHUS) and systemic lupus erythematous (SLE) [27], [28]. Therefore, the rs7542235 (*CFHR1–3Δ*) may contribute to hypertension risk by increasing inflammatory response, and rs2274700 (A473A) is likely to be in LD with other genetic variants that affect expression of CFH or binding capacity of CFH with CRP. Consistent with our observation that rs1061170 (Y402H) shows no significant association with blood pressure, a recent meta-analysis in more than 18,000 European individuals also found no evidence for association between rs1061170 (Y402H) and blood pressure and hypertension risk [29].

There is evidence that C-reactive protein can directly bind to CFH at both SCR-6/8 and SCR-16/20 sites, and consequently inhibit complement system activation and prevent inflammatory responses [8]. The sites of rs1061170 (Y402H) and rs2274700 (A473A) are both located at the CRP-binding sites in CFH [14], which may provide structural basis for the interaction between CRP and these genetic variants. Consistent with these notions, we found that the association between rs2274700 (A473A) and blood pressure was significant only in individuals with lower plasma CRP levels, suggesting that low CRP levels may be harmful to individuals carrying the blood-pressure-increasing allele of rs2274700 (A473A).

Taken together, our results suggest that common genetic variation in *CFH* and its related genes may contribute to variation in blood pressure and hypertension risk in Chinese Hans. However, further studies in additional independent cohorts are needed before a firm conclusion can be made.
